# Risk prediction of inappropriate implantable cardioverter-defibrillator therapy using machine learning

**DOI:** 10.1038/s41598-023-46095-y

**Published:** 2023-11-09

**Authors:** Ryo Tateishi, Makoto Suzuki, Masato Shimizu, Hiroshi Shimada, Takahiro Tsunoda, Hiroko Miyazaki, Yoshiki Misu, Yosuke Yamakami, Masao Yamaguchi, Nobutaka Kato, Ami Isshiki, Shigeki Kimura, Hiroyuki Fujii, Mitsuhiro Nishizaki, Tetsuo Sasano

**Affiliations:** 1 Department of Cardiology, Yokohama Minami Kyosai Hospital, 1-21-1 Mutsuura-Higashi, Kanazawa-ku, Yokohama, Japan; 2 Department of Cardiology, Odawara Cardiovascular Hospital, Odawara, Japan; 3 Department of Cardiology, Tokyo Medical and Dental University, Tokyo, Japan

**Keywords:** Cardiology, Medical research, Risk factors

## Abstract

We aimed to develop machine learning-based predictive models for identifying inappropriate implantable cardioverter-defibrillator (ICD) therapy. Our study included 182 consecutive cases (average age 62.2 ± 4.5 years, 169 men) and employed 14 non-deep learning models for prediction (hold-out method). These models utilized selected electrocardiogram parameters and clinical features collected after ICD implantation. From the feature importance analysis of the best ML model, we established easily calculable scores. Among the patients, 25 (13.7%) experienced inappropriate therapy, and we identified 16 significant predictors. Using recursive feature elimination with cross-validation, we reduced the features to six with high feature importance: history of atrial arrhythmia (Atr-arrhythm), ischemic cardiomyopathy (ICM), absence of diabetes mellitus (DM), lack of cardiac resynchronization therapy (CRT), V3 ST level at J point (V3 STJ), and V5 R-wave amplitudes (V5R amp). The extra-trees classifier yielded the highest area under receiver operating characteristics curve (AUROC; 0.869 on test data). Thus, the Cardi35 score was defined as [+ 5.5*Atr-arrhythm − 1.5*CRT + 1.0*V3STJ + 1.0*V5R − 1.0*ICM − 0.5*DM], which demonstrated a hazard ratio of 1.62 (P < 0.001). A cut-off value of the score + 5.5 showed high AUROC (0.826). The ML approach can yield a robust prediction model, and the Cardi35 score was a convenient predictor for inappropriate therapy.

## Introduction

Implantable cardioverter-defibrillators (ICDs) are highly effective in reducing mortality rates due to ventricular tachyarrhythmia among high-risk cardiac patients^1,2^. Several previous studies have reported prediction models for appropriate ICD therapy and prevention of sudden cardiac death with ICDs^3,4^. However, inappropriate ICD therapy occurs in 8–40% of patients with ICDs^5^. Inappropriate ICD therapy not only undermines the quality of life but also increases the risk of all-cause mortality^6^. Nearly 80% of inappropriate ICD shocks are caused by atrial fibrillation (AF) or supraventricular tachycardia^5^, and various predictors of inappropriate ICD shock, such as the absence of diabetes mellitus (DM), have also been indicated in previous studies^7^. Nevertheless, till date, no prediction model or scoring system has been able to accurately predict inappropriate ICD therapy.

Recently, machine learning (ML)-based studies have been published in the field of arrhythmia. Using several ML techniques, one such study attempted to predict future ventricular arrhythmia using ventricular signals^8^, and another attempted to estimate the risk of recurrence after AF ablation using data from cardiac computed tomography^9^. Moreover, the enhanced predictive capabilities of ML-based models over conventional clinical models have also been demonstrated. In one study, non-deep learning ML-based models, which combined 12-lead electrocardiogram (ECG) data and clinical features in a table data, further improved the prediction of patient outcome^10^. Concerning research on ICDs, a previous study attempted to predict electrical storms through machine learning using remote monitoring data^11^. However, to our knowledge, no studies have employed ML to predict inappropriate ICD therapy. We hypothesized that ML-based models trained using clinical features and non-invasive examinations, such as 12-lead ECG and ultrasound echocardiography (UCG) data, could provide superior prediction models. Furthermore, we attempted to extract the factors considered significant in the ML models to create a more practical scoring system for clinical application.

## Methods

### Patient population

This was a retrospective analysis of consecutive adult patients who underwent ICD implantation between January 2001 and December 2021 at a single center. To be included, patients were required to have undergone a 12-lead ECG within one week and echocardiography within 3 months before ICD implantation. Patients with inappropriate ICD therapy due to lead-related noise, subcutaneous ICDs, ventricular pacing prior to ICD implantation, and data deficits were excluded from the analysis. Initially, in this study, 201 patients underwent ICD implantation. Four patients were excluded due to lead-related noise, four due to subcutaneous ICDs, nine due to ventricular pacing prior to implantation, and ten due to missing data. Finally, 182 patients (62.2 ± 14.5 years, 169 men) were enrolled. The mean follow-up period was 2917 ± 2101 days. The study protocol adhered with the Declaration of Helsinki, and Yokohama Minami Kyosai Hospital Institutional Review Board approved this study with an opt-out option for the patients or proxy. The need for informed consent was waived by Yokohama Minami Kyosai Hospital Institutional Review Board owing to the retrospective nature of this study. The corresponding author had full access to all the data in the study and took responsibility for its integrity and data analysis.

### Patient background, clinical features, and non-invasive examinations

The patient background, extracted from electronic health records, included patient age at the time of ICD implantation, sex, and body mass index. Clinical comorbidities included hypertension, dyslipidemia, DM, chronic kidney disease, history of appropriate ICD therapy, atrial arrhythmia, and current smoking. We also checked for the worsening of heart failure and the new appearance of ischemic cardiomyopathy after ICD implantation. Underlying heart diseases were categorized as ischemic cardiomyopathy (ICM), dilated cardiomyopathy, hypertrophic cardiomyopathy, valvular heart disease, and channelopathy. The use of antiarrhythmic and cardioprotective drugs, and electrolyte status were also identified. If inappropriate ICD therapy was observed, the medication used by the patient and electrolyte status at that stage was included in the analysis; if no inappropriate ICD therapy was observed, the last follow-up medications were included. An automated system (ECAPs12c; Nihon-Koden, Tokyo, Japan) was used to measure the 12-lead ECG data, which were measured at the microvolt level, and the wave amplitude was defined as the absolute distance from the apex of each wave to the baseline. ECG variables were measured using 10-s waveforms. The rate-corrected QT interval (QTc) was calculated using the modified Framingham (ECAPs12C) formula (QTc = QT + [1000 – R-R]/7)^12^. The automatically measured data of < 100 ECG cases were excluded. All echocardiographic examinations were performed using the Vivid™ system (GE Healthcare, Chicago, Illinois, USA).

### ICD therapy

The ICD systems included systems that were manufactured by Biotronik (Berlin, Germany), Medtronic (Minneapolis, MN, USA), Boston Scientific (Marlborough, MA, USA), Sorin (Saluggia, Italy), and Abbott (Abbott Park, Illinois, USA). Since this study was a retrospective study, the choice of single- or dual-chamber devices, with or without LV leads, and ICD settings was according to the attending physician's judgment. The choice of single- or dual-chamber devices, cardiac resynchronization therapy (CRT), number of detection zones, and detection cycle length were included in the statistical analysis. Following previous studies, appropriate ICD therapy was defined as anti-tachycardia pacing or shock for ventricular arrhythmias, and inappropriate ICD therapy was for atrial arrhythmias^5,7^. Two board-certified Japanese Heart Rhythm Society members evaluated whether the therapy was appropriate or inappropriate.

### Statistical analysis

Fisher’s exact test was used to evaluate the differences in categorical variables. Continuous variables are displayed as the median value (interquartile range: 25–75% value), and the Student’s t-test or Mann–Whitney U test was used as appropriate. The cut-off values for continuous variables were calculated using receiver operating characteristic (ROC) curves at the point closest to the top-left corner. The Kaplan–Meier curve was used to evaluate the ML-based scoring systems. Statistical significance was set at p < 0.05. All statistical analyses were performed using EZR version 1.61 (Saitama Medical Center, Jichi Medical University, Saitama, Japan)^13^, which is a graphical user interface for R (The R Foundation for Statistical Computing, Vienna, Austria)^14^.

### Predictive model construction and validation by ML

To construct ML models, we adopted PyCaret 3.0 (https://pycaret.org), which is an open-source wrapper that is used over several ML libraries in Python in a low-code environment. We simultaneously built multiple non-deep learning models^15^. Statistically significant parameters were used in the creation of these ML models. In our ML analysis, we utilized hold-out method. The 182 cases were randomly divided into 65% for train-validation data (118 cases) and 35% for test data (64 cases). As a screening step, we compared fourteen ML methods on the 118 cases using the default hyperparameter for each ML model. or all procedures were performed on these 118 cases, tenfold cross-validation with 106 used as train data and 12 as validation data. Next, among the top 5 models with the highest area under receiver operating characteristics curve (AUROC), various optimizations were applied. For each model in the top 5 ML models, we primarily executed the following five optimization procedures. Due to the dataset’s imbalance, when evaluating performance, our performance evaluation was centered on AUROC and F1-score, which represents the harmonic mean of precision (= positive predictive value) and recall (= sensitivity). Models with an F1-score ≤ 0.30 were excluded from consideration.

1. Five oversampling methods: ADASYN, BorderlineSMOTE, RandomOverSampler, SMOTE, SVMSMOTE.
2. Search the best number of features: Boruta method, Relief filter method (using scikit-rebate), Recursive feature elimination with cross validation (RFECV).^16^
3. Hyperparameter tuning by Optuna method (on some models).^17^
4. Ensemble of boosting and bagging.
5. Blend of two models.

In all tuned models, all the 118 cases were utilized to build the last models. And the diagnostic performance was evaluated on the test data (64 cases) by the last models. The details of the five last models were described in Supplemental Table S1.

The feature importance was calculated in decision tree models, which was obtained by summing up the Gini impurity reductions for that feature across all the nodes in which it is used for splitting^18^. The feature importance of the models was ranked to estimate the contribution of the predictors to the ML model. In addition to feature importance, the SHAP (SHapley Additive exPlanations) method was introduced for evaluation of the features^19^. The SHAP method theory is based on “the game optimal Shapley values”, and the summary plot of the SHAP combines feature importance with feature effects. Different color points indicate patients who did not receive inappropriate ICD therapy (blue point, in Fig. 2) and those who did (red point, in Fig. 2). The Shapley value of each feature is displayed on the x-axis (defined as the SHAP value), where a large (right side) value indicates a positive contribution to the model. The SHAP method is briefly explained in Supplemental Fig. S1.

Finally, we extracted important features from both feature importance and SHAP findings. Important factors were assigned a score based on the feature importance score and the mean absolute SHAP value and were summed and multiplied by 10. To simplify the calculation, we assigned the score of each factor the closest value in increments of ± 0.5 from 0. We added “ + ” to a score for each factor when a positive correlation with inappropriate ICD therapy was found and added “–” when a negative correlation was found.

## Results

### Baseline patient characteristics and inappropriate ICD therapy

A total of 25 patients (13.7%) received inappropriate ICD therapy. Baseline characteristics were displayed in Table 1, and ECG and UCG data were shown in Table 2. There were no significant differences of age and follow-up period between the two groups (inappropriate ICD therapy group and no therapy group). Sixteen statistically significant factors were extracted, which included the absence of DM, history of atrial arrhythmia, lack of CRT, ICM, and verapamil administration. The two groups had no difference in the number of ICD detection zones and chambers or maximum detection cycle length. Of the 25 patients who received inappropriate ICD therapy, 13 (52.0%) received shock therapy and 15 (60.0%) experienced anti-tachycardia pacing; three patients experienced both. Inappropriate ICD therapy was caused by AF in 16 patients (64.0%), supraventricular tachycardia, including atrial flutter, in six patients (24.0%), and sinus tachycardia in three patients (12.0%). Although this study incorporated both primary and secondary prevention, the median LVEF was low (38%). However, the two groups showed no significant differences in echocardiographic findings. Five factors of baseline characteristics (in Table 1) and 11 parameters of ECG (in Table 2) showed significant differences, and were incorporated into the ML model. All comprehensive ECG findings are listed in Supplemental Table S2.

**Table 1:** Baseline characteristics of the population.

	All patients (n = 182)	Inappropriate therapy ( +) (n = 25)	Inappropriate therapy (−) (n = 157)	p value
Follow-up duration (days)	2917.2 (± 2101.1)	3040.9 (± 1792.5)	2897.5(± 2150.5)	0.752
Patient background				
Male (n)	150 (82.4%)	20 (80.0%)	130 (82.8%)	0.778
Age (years)	62.3 (± 14.5)	63.2 (± 11.9)	62.1 (± 14.9)	0.736
Body mass index (kg/m^2^)	22.8 [19.6–25.3]	22.8 [19.6–24.6]	22.9 [19.6–25.4]	0.573
Comorbidities				
Hypertension (n)	65 (35.7%)	7 (28.0%)	58 (36.9%)	0.502
Diabetes (n)	73 (40.1%)	5 (20.0%)	66 (42.0%)	**0.046**
Dyslipidemia (n)	56 (30.8%)	8 (32.0%)	48 (30.6%)	1.000
Chronic kidney disease (n)	69 (37.9%)	11 (44.0%)	58 (36.9%)	0.513
Appropriate ICD therapy (n)	50 (24.7%)	4 (16.0%)	49 (31.2%)	0.156
History of atrial arrhythmia (n)	77 (42.3%)	23 (92.0%)	54 (34.4%)	** < 0.001**
Current smoking (n)	64 (35.2%)	7 (28.0%)	57 (36.3%)	0.503
Cardiac event after implantation of ICD				
Aggravation of heart failure (n)	49 (26.9%)	5 (20.0%)	44 (28.0%)	0.475
New appearance of ischemic cardiomyopathy (n)	23 (12.6%)	1 (4.0%)	22 (14.1%)	0.209
Underlying heart disease				
Ischemic cardiomyopathy (n)	72 (39.6%)	4 (16.0%)	68 (43.3%)	**0.014**
Dilated cardiomyopathy (n)	40 (22.0%)	9 (36.0%)	31 (19.7%)	0.115
Hypertrophic cardiomyopathy (n)	13 (7.1%)	3 (12.0%)	10 (6.4%)	0.393
Valvular heart disease (n)	5 (2.7%)	2 (8.0%)	3 (1.9%)	0.140
Brugada syndrome (n)	24 (13.2%)	3 (12.0%)	21 (13.4%)	1.000
Long QT syndrome (n)	3 (1.6%)	0 (0.0%)	3 (1.9%)	1.000
ARVC (n)	1 (0.1%)	0 (0.0%)	1 (0.1%)	1.000
IVF/IVT (n)	8 (4.4%)	2 (8.0%)	6 (3.8%)	0.302
Others (n)	16 (8.8%)	2 (8.0%)	14 (8.9%)	1.000
Medication				
Class Ia (n)	7 (3.8%)	0 (0.0%)	7 (4.5%)	0.596
Class Ib (n)	17 (9.3%)	4 (16.0%)	13 (8.3%)	0.260
Class Ic (n)	2 (1.1%)	0 (0.0%)	2 (1.3%)	1.000
Amiodarone (n)	42 (23.1%)	7 (28.0%)	35 (22.3%)	0.609
β-blocker (n)	121 (66.5%)	17 (68.0%)	104 (66.2%)	1.000
Bepricol (n)	11 (6.0%)	1 (4.0%)	10 (6.4%)	1.000
Verapamil (n)	7 (3.8%)	4 (16.0%)	3 (1.9%)	**0.007**
ACE-I/ARB (n)	52 (28.6%)	3 (12.0%)	49 (31.2%)	0.057
MRA (n)	46 (25.3%)	6 (24.0%)	40 (25.5%)	1.000
Electrolyte status				
Sodium (mEq/L)	139.4 (± 3.4)	139.3 (± 3.3)	139.4 (± 3.4)	0.887
Potassium (mEq/L)	4.3 (± 0.5)	4.3 (± 0.5)	4.2 (± 0.6)	0.439
Chlorine (mEq/L)	108.7 (± 7.4)	104.1 (± 3.6)	109.5 (± 8.0)	0.735
ICD programing settings				
Dual-chamber device (n)	109 (59.9%)	17 (68.0%)	92 (58.6%)	0.510
Ventricular pacing rate (%)	0.1 [0.0–5.0]	0.2 [0.1–1.0]	0.1 [0.0–6.0]	0.978
CRT-D (n)	36 (19.8%)	1 (4.0%)	35 (22.3%)	**0.032**
Detection cycle length (ms)	333.0 [320.0–373.8]	330.0 [320.0–370.0]	355.0 [320.0–400.0]	0.301
ICD detection zones				0.185
1 zone (%)	95 (52.2%)	9 (36.0%)	86 (54.8%)	
2 zones (%)	67 (36.8%)	13 (52.0%)	54 (34.4%)	
3 zones (%)	20 (11.0%)	3 (12.0%)	17 (10.8%)	

Values are presented as numbers, mean ± SD, or median (interquartile range). Categorical variables were compared using Fisher’s exact test; continuous variables were compared using Student’s t-test or Mann–Whitney U test if data were not normally distributed.

*ACE-I* angiotensin-converting enzyme inhibitor, *ARB* angiotensin receptor blocker, *ARVC* arrhythmogenic right ventricular cardiomyopathy, *CRT-D* cardiac resynchronization therapy, *ICD* implantable cardioverter-defibrillator, *IVT* idiopathic ventricular tachycardia, *IVF* idiopathic ventricular fibrillation, *MRA* mineralocorticoid receptor antagonists.

Significant values are in bold.

**Table 2:** Non-invasive examination.

	All patients (n = 182)	Inappropriate therapy (+) (n = 25)	Inappropriate therapy (−) (n = 157)	p value
Echocardiography				
LAD (mm)	40.8 (± 8.42)	41.1 (± 7.33)	40.8 (± 8.61)	0.850
LVDd (mm)	57.0 (± 9.88)	56.0 (± 8.55)	57.1 (± 10.1)	0.600
LVDs (mm)	44.5 (± 12.3)	43.5 (± 11.4)	44.6 (± 12.5)	0.692
IVS (mm)	9.7 [8.4–10.9]	9.8 [8.2–11.1]	9.7 [8.5–10.9]	0.595
LVPW (mm)	9.5 [8.4–10.6]	9.3 [8.0–11.0]	9.5 [8.5–10.5]	0.731
LVEF (%)	38.0 [31.0–55.0]	37.0 [30.0–52.0]	38.0 [31.0–55.0]	0.980
Significant factors of ECG characteristics				
II T-wave amplitude (μV)	119.6 (± 190.3)	26.7 (± 232.4)	134.0 (± 179.6)	**0.012**
aVR T-wave amplitude (μV)	−94.2 (± 144.1)	−23.9 (± 165.9)	−105.8 (± 137.4)	**0.013**
V3 ST J (μV)	45.0 [5.0–85.0]	15.0 [−30.0 to 40.0]	50.0 [5.0–95.0]	**0.001**
V3 ST MID (μV)	120.0 [40.0–180.0]	60.0 [35.0–115.0]	130.0 [45.0–185.0]	**0.019**
V4 R-wave amplitude (μV)	1158.3 (± 872.4)	1781.2 (± 1119.6)	1054.5 (± 781.5)	** < 0.001**
V4 QRS area (40 ms μV)	171.5 [−608.0 to 574.8]	394.0 [37.0–820.0]	133.0 [−669.0 to 497.0]	**0.028**
V4 ST J (μV)	−2.5 [−43.8 to 40.0]	−40.0 [−90.0 to 10.0]	0.0 [−35.0 to 45.0]	**0.004**
V4 ST MID (μV)	55.0 [0.0–115.0]	15.0 [−50.0 to 65.0]	55.0 [5.0–120.0]	**0.017**
V5 R-wave amplitude (μV)	1403.1 (± 920.2)	2098.6 (± 1145.8)	1289.5 (± 828.3)	** < 0.001**
V5 QRS area (40 ms μV)	171.5 [−608.0 to 574.8]	805.0 [565.0–1489.0]	516.0 [75.0–940.0]	**0.011**
V6 R-wave amplitude (μV)	1139.9 (± 686.6)	1539.8 (± 904.6)	1074.9 (± 624.3)	**0.002**

Values are presented as numbers, mean ± SD, or median (interquartile range: 25%, 75%). Continuous Numeric variables were analyzed using Student’s t-test or Mann–Whitney U test if the data were not normally distributed.

*IVS* interventricular septum, *LAD* left atrial diameter, *LVDd* left ventricular end-diastolic diameter, *LVDs *left ventricular end-systolic diameter, *LVEF* left ventricular ejection fraction, *LVPW* left ventricular posterior wall.

Significant values are in bold.

### ML prediction model construction and evaluation

**Figure 1:**
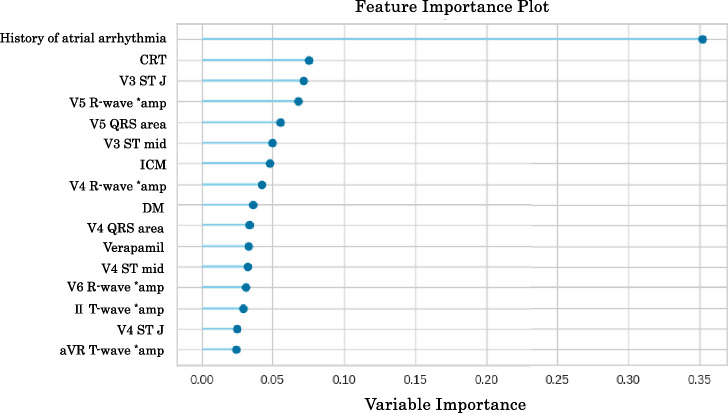
Feature importance of extra trees classifier on train-validation data. Feature importance on train-validation data in predicting inappropriate implantable cardioverter-defibrillator (ICD) therapy using the extra-trees classifier. The feature importance was calculated in decision tree models, which was obtained by summing up the Gini impurity reductions for that feature across all the nodes in which it is used for splitting. The features were sorted in order to value of the importance. * amp amplitude.

**Figure 2:**
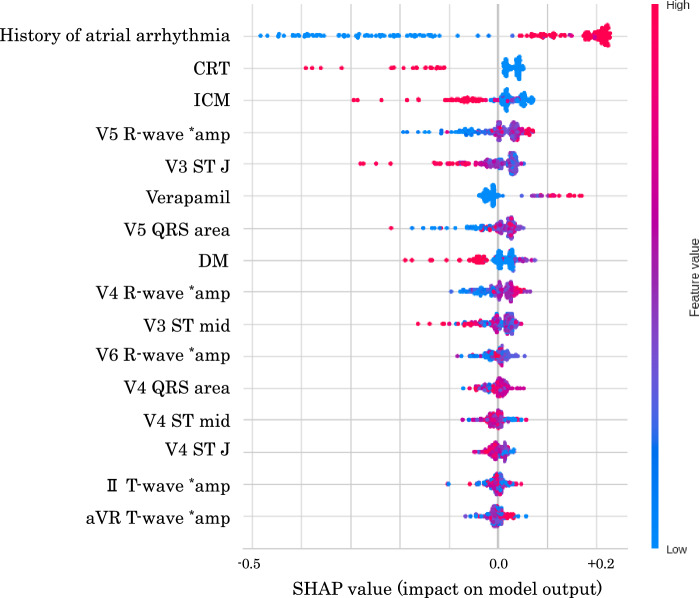
SHAP method on extra trees classifier. Interpretation of feature importance using the SHapley Additive exPlanation (SHAP) method for Extra trees classifier on the train-validation data, which is based on “the game optimal Shapley values”. Blue color points indicate patients who did not receive inappropriate ICD therapy, and those who did (red points). The Shapley value of each feature is displayed on the x-axis (defined as the SHAP value), where a large (right side) value indicates a positive contribution to the model. The six features showing the highest absolute SHAP value were a history of atrial arrhythmia, cardiac resynchronization therapy (CRT), ischemic cardiomyopathy (ICM), V5 R-wave amplitudes, V3 ST level at J point, and verapamil administration. The interpretation of SHAP method is briefly explained in Supplemental file S2. * amp amplitude.

The ML model incorporated the statistically significant 16 predictors using PyCaret. Through screening analysis, we identified five ML models with high AUROC values (Extra trees classifier, Gradient boosting Classifier, CatBoost classifier, Extreme gradient boosting, and Light gradient boosting machine, shown in Table 3). The five models were fine-tuned to achieve higher AUROC using tenfold cross-validation on 118 train-validation data, and their final performances was evaluated on 64 test data. Ultimately, we selected the Extra-trees classifier (model_ET) as the best model. This classifier is an exceptional ensemble learning method that employs numerous random decision trees to create a majority vote-like system^16^. In the model_ET, the 16 predictors (referred to as features) were reduced to the optimal number 6 using RFECV with high feature importance (the history of atrial arrhythmia, CRT, ischemic cardiomyopathy (ICM), DM, and V3 ST level at J point (V3STJ), and V5 R-wave amplitudes (V5R amp). The model_ET achieved an AUROC of 0.869 and the F1-score was 0.533 on test data. The feature importance of the model_ET was depicted in Fig. 1. We also evaluated the importance of the features by another procedure using the SHAP method (Fig. 2). The six features extracted by RFECV procedure showed high importance in both methods. Because the ML-based model verified two numeric variables (V3 STJ and V5R amp) as significant, we performed ROC curve analysis for them to determine these cut-off values on the train-validation data. The cut-off value of the V3 STJ was 20 μV, and that of V5R amp was 1400 μV with relatively high AUROC (V3 STJ: 0.699, V5R amp: 0.717, respectively). The ROC curves of them are shown in Supplemental Fig. S2.

**Table 3:** Results of machine learning predictive models built by PyCaret.

	AUROC	ACC	Spec	Sens (recall)	PPV (prec.)	NPV	F1
Train-validation							
Extra trees classifier	0.891	0.836	0.873	0.650	0.425	0.942	0.487
CatBoost classifier	0.875	0.820	0.861	0.550	0.325	0.930	0.397
Gradient boosting classifier	0.846	0.871	0.901	0.650	0.533	0.948	0.577
Light gradient boosting machine	0.860	0.769	0.792	0.600	0.300	0.936	0.387
Extreme gradient boosting	0.841	0.821	0.871	0.500	0.342	0.921	0.380
Test							
Extra trees classifier	0.869	0.781	0.764	0.889	0.381	0.977	0.533
CatBoost classifier	0.848	0.766	0.764	0.778	0.350	0.955	0.483
Gradient boosting classifier	0.830	0.813	0.873	0.444	0.364	0.906	0.400
Light gradient boosting machine	0.815	0.813	0.873	0.444	0.364	0.906	0.400
Extreme gradient boosting	0.779	0.797	0.855	0.444	0.333	0.904	0.381

The upper table shows the results top 5 area under the receiver operating characteristic curve (AUROC) of screening on train-validation data (after tuning), and the lower table demonstrates the diagnostic performance of the tuned models on the test data. The results of upper table were selected to produce the highest AUROC on the test data, and the data was sorted by AUROC on the test data. All results of first screening for the models on train-validation data were described in Supplemental Table S4. The details of the top five models were explained in Supplemental Table S1.

*ACC* accuracy, *AUROC* area under the receiver operating characteristic curve, *F1* F1-score (harmonic mean of precision and recall), *NPV* negative predictive value, *PPV* positive predictive value (identical to precision), *Sens* sensitivity (identical to recall), *Spec* specificity.

### Scoring system

**Figure 3:**
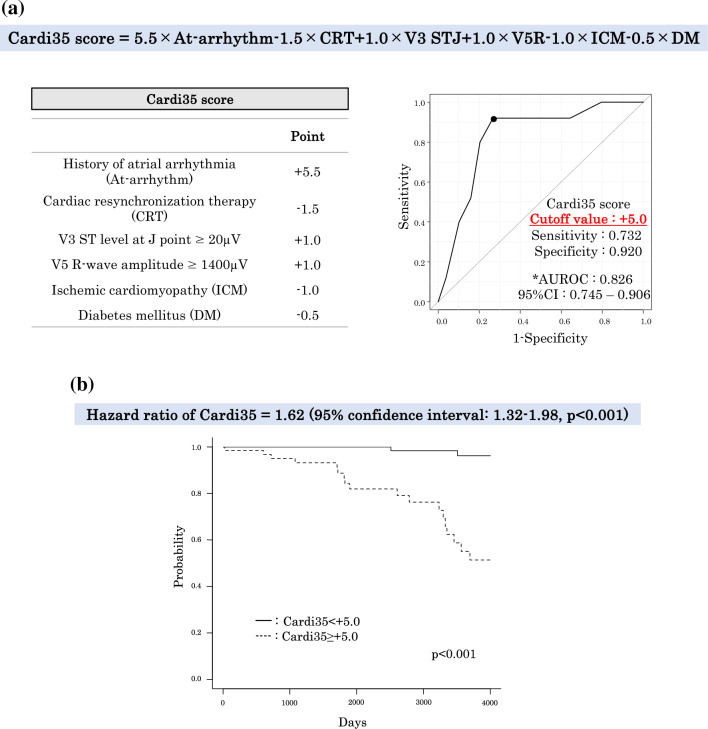
Cardi35 score for predicting inappropriate ICD therapy. (**a**) Definition of Cardi35 score and cut-off value of receiver operating characteristic curve analysis. On train-validation data, the predictive scores, calculated using this linear combination formula, was referred to as the Cardi35 score, which includes *C*RT, atrial *Ar*rhythmia, *D*M, *I*CM, and V*3* STJ and V*5*R amp. The six features were selected from the search the best number of features: Recursive feature elimination with cross validation method (RFECV). The score was weighted coefficient (= “point” in the table) derived from evaluation of features by Extra trees classifier on train-validation data. The score takes the values from − 3.0 to + 7.5. The cut-off value was + 5.0, with sensitivity and specificity of 0.732 and 0.920, respectively. The AUROC was 0.826 and the 95% confidence interval (CI) was 0.745–0.906. *Asterisk* area under the receiver operating characteristic curve. (**b**) Validation of Cardi35 score on the test data. The prognostic value of Cardi35 was validated on the test data. Hazard ratio of this score was calculated as 1.62 by Cox regression analysis (95% confidence interval: 1.32–1.98, p < 0.001). Furthermore, Kaplan–Meier curve analysis demonstrates a significantly worse inappropriate therapy outcome in the group with a Cardi35 score ≥  + 5.0 compared to the group with a score <  + 5.0 (log-rank: p < 0.001).

Based on the feature importance score and SHAP value from the train-validation data using model_ET, we developed a scoring system for predicting inappropriate ICD therapy. The scoring system relied on six specific features that were selected by RFECV procedure shown in Supplemental Fig. S3. The definition of the score was illustrated in Fig. 3, and further details about its foundation could be found in Supplemental Table S3. In brief, the score was computed by summing the feature importance score and the mean SHAP value for each feature. The value were as follows: 0.563 for a history of atrial arrhythmia, 0.135 for CRT, 0,112 for V5R amp, 0,103 for V3 STJ, 0.095 for ICM, and 0.067 for DM. These values were then multiplied by 10 and rounded to the nearest number in 0.5 increments for easier calculation of scores. Additionally, positive and negative correlations were considered, with " + " and "−" signs. This final sum resulted in the following assignments: + 5.5 points for a history of atrial arrhythmia, − 1.5 for CRT, + 1.0 for V3 STJ ≥ 20 μV and V5R amp ≥ 1400 μV, − 1.0 for ICM, and − 0.5 for DM.

The predictive scores, calculated using this linear combination formula, was referred to as the *Cardi35* score, which includes *C*RT, atrial *Ar*rhythmia, *D*M, *I*CM, and V*3* STJ and V*5*R amp, as shown in Fig. 3(a). ROC curve analysis demonstrated + 5.0 as cut-off value on the train-validation data, and the AUROC of the score was 0.826 (specificity: 0.920, sensitivity: 0.732). The prognostic value of Cardi35 was validated on the test data. Hazard ratio of this score was calculated as 1.62 by Cox regression analysis (95% confidence interval: 1.32–1.98, p < 0.001). Furthermore, Kaplan–Meier curve analysis demonstrates a significantly worse inappropriate therapy outcome in the group with a Cardi35 score ≥  + 5.0 compared to the group with a score <  + 5.0 (log-rank: p < 0.001), as depicted in Fig. 3(b).

## Discussion

We developed an ML-based model that achieved an AUROC of 0.869 on the test data for predicting inappropriate ICD therapy. While the adverse consequences of inappropriate ICD therapy are well-known, there are limited data regarding its prediction. Notably, several of the factors identified as significant in our study align with findings from prior research. For instance, atrial arrhythmias have consistently been identified as a common trigger for inappropriate ICD therapy^5^. The absence of DM as a significant factor might be attributed to the possibility that patients with DM could be more immobile and might exhibit autonomic dysfunction^7^. Additionally, individuals with ICM are less likely to experience inappropriate ICD therapy compared to those without ICM. A previous study demonstrated that inappropriate ICD shocks triggered by atrial arrhythmias were more prevalent in patients without ICM than in those with ICM^20^. Furthermore, it is established that appropriate settings can effectively suppress atrial fibrillation (AF), which could account for the decreased occurrence of inappropriate ICD therapy in patients with a history of cardiac resynchronization therapy (CRT)^21^. However, no previous study has demonstrated a clear link between V3 ST segment level at the J point and V5 R-wave amplitudes with inappropriate ICD therapy. We have considered two plausible theories to explain this association. Firstly, the V5R amplitude may be correlated with left ventricular hypertrophy, which in turn could potentially trigger atrial fibrillation (AF)^22^. The inclusion of V5R amplitude as a diagnostic criterion for left ventricular hypertrophy on an electrocardiogram (ECG) lends support to this hypothesis. Secondly, the V3 ST segment level at the J point, a marker associated with Brugada syndrome, might influence the occurrence of atrial arrhythmias. In a prior study, inappropriate ICD therapy in Brugada syndrome patients and its relationship with atrial arrhythmias were reported^23^.

Machine learning applied to 12-lead ECG data has primarily been developed using deep learning techniques, which utilize numeric data from ECGs represented as time–voltage plots in CSV format^24^. While deep learning offers the advantage of achieving high accuracy, it demands a substantial amount of data and high-performance computing resources to attain superior diagnostic performance. Notably, ECG data in CSV format is not readily available in general hospitals and clinics, which can limit the generalizability of results and models. Conversely, non-deep learning methods, as employed in the present study, may not reach the same level of performance. However, they do offer the advantage of being able to construct predictive models with relative ease, even with a smaller number of samples, while maintaining good robustness and reproducibility^12^.

While various predictors, including patient background, have been explored in previous research^25^, the development of predictive models has remained a challenge. Employing machine learning (ML), we have introduced the Cardi35 score, serving as a predictive model with an AUROC of 0.826. This performance is comparable to that of complex ML models, which often surpass the accuracy of clinical scoring systems. This difference is evident when considering established clinical scores for atrial fibrillation (AF) ablation, where AUROCs typically range from 0.55 to 0.65, and models achieving an AUROC of 0.75 are considered rare^26,27^. In the context of ML models, Shade et al. achieved an AUROC of 0.82 using contrast-enhanced MRI^28^, and Tang et al. obtained an AUROC of 0.86 by utilizing an ML model that incorporated ECG and intracardiac ECG data^10^. Our study has demonstrated an AUROC that is comparable, indicating sufficient accuracy. We believe that this ML model can be effectively employed as a scoring system for predicting inappropriate ICD therapy.

The pivotal aspect of the Cardi35 score is that it does not designate any high-risk factors unless there is a history of atrial arrhythmia. This observation may stem from the inherent difficulty in predicting AF and supraventricular tachycardia through non-invasive examinations. Previous studies have explored the use of echocardiography and deep learning ECG to forecast AF^24,29,30^. However, these studies either achieved an AUROC of less than 0.7 or relied on vast amounts of data (over 450,000 datasets). Therefore, in a study of this scale, predicting atrial arrhythmia in patients without a prior history of such conditions would have presented a formidable challenge. Nonetheless, it is valuable to assess whether patients with a history of atrial arrhythmia are at elevated risk. A recent study has presented discouraging long-term prognosis data concerning ICD implantation for primary prevention^31^. Consequently, it becomes pivotal to evaluate the presence of diabetes mellitus (DM), cardiac resynchronization therapy (CRT), and V5R amplitude in individuals with a history of atrial arrhythmia, especially in those without ischemic cardiomyopathy (ICM), to gauge the potential risk of inappropriate ICD therapy. Considering the accuracy of this score, it could aid in determining the necessity for an aggressive rhythm control strategy in high-risk patients or assessing whether ICD implantation should be considered on a case-by-case basis.

This study has several limitations. Firstly, it involved a small cohort, and the findings have not undergone external validation. While we were able to showcase high diagnostic performance through the hold-out method, the ML model which constructed with all 182 cases was not assessed externally. Both the model and our Cardi35 score require validation with additional external datasets. Secondly, due to the retrospective nature of the study, there was limited available information. For instance, factors such as the left atrial volume index^29^ and left ventricular diastolic dysfunction are known to be associated with atrial fibrillation (AF)^32^. However, these data were frequently absent from older datasets, and the outcomes might have differed if these variables had been included in the analysis. Thirdly, there is currently no standardized method for generating clinical scores from ML models. In this study, we devised scores using feature importance scores and SHAP values, and the number of selected factors was determined with consideration for practical clinical application. However, this decision was somewhat arbitrary, and the possibility of a more optimal scoring system cannot be dismissed. Despite these limitations, the Cardi35 score maintains a high level of accuracy, underscoring its value in clinical practice.

## Conclusions

Our study has demonstrated the effectiveness of the machine learning (ML) approach in developing resilient prediction models for inappropriate ICD therapy. The Cardi35 score, derived from this model, offers a convenient means of assessing whether an aggressive rhythm control strategy should be pursued or if ICD implantation should be considered for specific patients.

### Supplementary Information

Supplementary Information.

## Data Availability

The datasets used and/or analyzed during the current study are available from the corresponding author on reasonable request.
