# Supplementary material for: Risk prediction of inappropriate implantable cardioverter-defibrillator therapy using machine learning
Source: Sci Rep. 2023 Nov 9;13:19586. doi: 10.1038/s41598-023-46095-y (PMC10638417; doi:10.1038/s41598-023-46095-y)
Supplement: Supplementary file 1 — Supplementary Information. [file 41598_2023_46095_MOESM1_ESM.docx]

**SUPPLEMENTAL MATERIAL**

**Supplemental Figure S1. Interpretation of SHAP method**

The SHapley Additive exPlanation (SHAP) method is based on the concepts of game theory. It can be used to explain the predictions of machine learning models by calculating the contribution of each feature to the prediction. In a game, when each player distributes to it solitarily or cooperatively, a total reward is calculated based on each coalition of all players. The SHAP value is the importance of each player to the overall cooperation, which is an expected payoff. SHAP scattergram of ensemble learning indicates two characteristics of features, the contribution of each feature and the correlation of objective variable and feature. In the figure, wide SHAP (A and B) indicates a high contribution. On the other hand, narrow SHAP (C and D) shows a low contribution. A and C demonstrate positive correlations, and B has a negative correlation. * amplitude.

**Supplemental Figure S2. *Receiver operating characteristic* *curve of V3 ST level at J point and V5 R-wave amplitude***

The cut-off value was determined as the point on the curve with a minimum distance from the left-upper corner on train-validation data. As a result, the cut-off value of V3 ST level at J point was 20 μV and that of V5 R-wave amplitude was 1400 μV. The sensitivity and specificity of V3 ST level at J point were 0.707 and 0.640, and those of V5 R-wave amplitude were 0.720 and 0.614, respectively. The area under the receiver operating characteristic curve of V3 ST level at J point was 0.699 (95% confidence interval [CI], (0.602-0.796) and that of V5 R-wave amplitude was 0.717 (95% CI, 0.611-0.823).

**Supplemental Figure S3. *Method to decide the number of features (RFECV by PyCaret)***

As the method to decide the number of features on train-validation data, we adopted RFECV (recursive feature elimination with cross validation) method was adopted. The result was demonstrated as the following graph. X-axis indicates the number of features and Y-axis shows cross validation score (ratio of correct classification).

In extra trees classifier, when the number of feature increases over five, the score reached to plateau. We investigated the area under receiver operating characteristics curve (AUROC) using the number of 5/6/7/8. As the results, the number which demonstrated the highest AUROC was decided as six.

**Supplemental Table S1.** Final machine learning models

Selected five machine learning (ML) models were tuned to get higher area under receiver operating characteristics curve (AUROC). The results that demonstrated the highest AUROC were shown in Table 3, and the details of the final five models were explained as the following upper table. The best model was Extra trees classifier (model_ET), whose hyperparameters was shown in the following lower table.

|  | **Oversampling** | **Reduction of features** | **Optimization** | **Other tuning** |
| --- | --- | --- | --- | --- |
| **Extra trees classifier** | ADASYN | RFECV (6) | None | Optuna |
| **Gradient Boosting Classifier** | ADASYN | ReliefF (4) | AUROC | None |
| **CatBoost Classifier** | ADASYN | ReliefF (4) | AUROC | Ensemble  (Bagging&Boosting) |
| **Extreme Gradient Boosting** | SMOTE | ReliefF (4) | AUROC | Optuna |
| **Light Gradient Boosting Machine** | SMOTE | ReliefF (4) | None | None |

RFECV (recursive feature elimination with cross validation) methods extracted 6 parameters (History of atrial arrhythmia, cardiac resynchronization therapy, ischemic cardiomyopathy, diabetes mellitus, V3 ST level at J point, and V5 R-wave amplitude).

Relief filter method extracted 4 parameters (History of atrial arrhythmia, ischemic cardiomyopathy, diabetes mellitus, V5 R-wave amplitude).

| **Hyperparameters of model_ET** | |
| --- | --- |
| **Index of model_ET** | **Parameters** |
| **Number of features** | 6 |
| **bootstrap** | False |
| **ccp_alpha** | 0.0 |
| **class_weight** | None |
| **criterion** | Entropy |
| **max_depth** | 7 |
| **max_features** | 0.959387836948285 |
| **max_leaf_nodes** | None |
| **max_samples** | None |
| **min_impurity_decrease** | 3.797437735509758e-07 |
| **min_samples_leaf** | 3 |
| **min_samples_split** | 5 |
| **min_weight_fraction_leaf** | 0.0 |
| **n_estimators** | 44 |
| **n_jobs** | -1 |
| **oob_score** | False |
| **random_state** | 4 |
| **verbose** | 0 |
| **warm_start** | False |

**Supplemental Table S2**. All items related to the electrocardiogram

Data from less than 100 cases were excluded. Numeric variables are shown as mean±SD or median (interquartile range: 25%, 75%), and the Student's t-test or Mann–Whitney U test was performed as appropriate. * ST junction; † rate-corrected QT interval.

|  | **All subjects** | **Inappropriate therapy (+)** | **Inappropriate therapy (-)** | **p value** |
| --- | --- | --- | --- | --- |
|  | **(n=182)** | **(n=25)** | **(n=157)** |  |
| Heart rate (bpm) | 68.4 (±15.4) | 73.9 (±22.0) | 67.5 (±13.9) | 0.055 |
| P-wave axis (degree) | 46.3 (±36.0) | 49.3 (±37.3) | -45.9 (±35.9) | 0.701 |
| PQ interval (msec) | 190.0 [166.0–216.0] | 184.0 [170.0–212.0] | 190.0 [166.0–216.0] | 0.486 |
| QRS axis (degree) | 29.2 (±60.1) | 37.9 (±51.9) | 27.8 (±61.3) | 0.434 |
| QRS duration (msec) | 109.0 [98.0–126.0] | 100.0 [92.0–118.0] | 110.0 [98.0–126.0] | 0.159 |
| T-wave axis (degree) | 68.0 [30.3–108.0] | 62.0 [34.0–155.0] | 68.0 [30.0–106.0] | 0.789 |
| QT interval (msec) | 426.5 (±45.1) | 421.4 (±47.5) | 427.3 (±44.8) | 0.543 |
| ^†^QTc (msec) | 439.2 (±33.9) | 442.5 (±28.8) | 438.7 (±34.7) | 0.604 |
| Ⅰ P-wave amplitude (μV) | 65.0 [45.0–80.0] | 57.5 [40.0–70.0] | 65.0 [45.0–80.0] | 0.407 |
| Ⅰ QRS area(40 ms＊μV) | 294.6 (±350.6) | 315.5 (±404.8) | 291.3 (±342.6) | 0.749 |
| Ⅰ R-wave amplitude (μV) | 549.8 (±307.1) | 567.0 (±291.5) | 547.0 (±310.4) | 0.764 |
| Ⅰ R-wave duration (msec) | 56.0 [41.0–72.0] | 54.0 [42.0–68.0] | 56.0 [40.5–72.0] | 0.993 |
| Ⅰ S-wave amplitude (μV) | 120.0 [80.0–215.0] | 115.0 [100.0–240.0] | 122.5 [75.0–215.0] | 0.615 |
| Ⅰ S-wave duration (msec) | 38.0 [28.0–58.0] | 30.0 [27.0–57.0] | 38.0 [28.0–57.5] | 0.656 |
| Ⅰ ^*^STJ (μV) | -10.0 [-25.0–13.8] | -10.0 [-25.0–15.0] | -10.0 [-25.0–10.0] | 0.522 |
| Ⅰ ST MID (μV) | 0.5 (±41.8) | -6.4 (±43.7) | 1.6 (±41.5) | 0.378 |
| Ⅰ ST END (μV) | 5.0 [-25.0–43.8] | 0.0 [-25.0–45.0] | 5.0 [-25.0–40.0] | 0.521 |
| Ⅰ T-wave amplitude (μV) | 63.2 (±134.1) | 52.5 (±131.8) | 64.7 (±134.8) | 0.705 |
| Ⅰ T-wave amplitude modulation (μV) | 16.0 (±110.1) | 6.0 (±112.1) | 17.4 (±110.1) | 0.656 |
| Ⅰ Ventricular activation time (ms) | 46.0 [40.0–58.0] | 44.0 [42.0–54.0] | 46.0 [40.0–58.0] | 0.682 |
| Ⅱ P-wave amplitude (μV) | 90.0 [65.0–120.0] | 75.0 [50.0–110.0] | 90.0 [65.0–120.0] | 0.223 |
| Ⅱ QRS area(40 ms＊μV) | 299.0 [9.0–512.8] | 269.0 [66.0–549.0] | 302.0 [9.0–499.0] | 0.719 |
| Ⅱ R-wave amplitude (μV) | 602.8 (±370.8) | 641.0 (±411.0) | 596.8 (±365.2) | 0.589 |
| Ⅱ R-wave duration (msec) | 59.2 (±25.4) | 56.4 (±20.3) | 59.7 (±26.1) | 0.562 |
| Ⅱ S-wave amplitude (μV) | 240.0 [125.0–388.8] | 200.0 [93.8–328.8] | 247.5 [128.8–401.3] | 0.271 |
| Ⅱ S-wave duration (msec) | 47.9 (±26.8) | 42.2 (±25.5) | 49.0 (±27.0) | 0.328 |
| Ⅱ ^*^STJ (μV) | -10.0 [-40.0–20.0] | -10.0 [-60.0–15.0] | -10.0 [-40.0–20.0] | 0.305 |
| Ⅱ ST MID (μV) | 13.0 (±62.1) | -7.0 (±63.3) | 16.1 (±61.5) | 0.084 |
| Ⅱ ST END (μV) | 25.0 [-20.0–75.0] | 10.0 [-20.0–70.0] | 25.0 [-20.0–75.0] | 0.423 |
| Ⅱ T-wave amplitude (μV) | 119.6 (±190.3) | 26.7 (±232.4) | 134.0 (±179.6) | **0.012** |
| Ⅱ T-wave amplitude modulation (μV) | 60.0 [-52.0–143.0] | 46.5 [-128.8–102.5] | 65.0 [-22.0–145.0] | 0.088 |
| Ⅱ Ventricular activation time (ms) | 44.0 [38.0–52.0] | 44.0 [37.5–50.0] | 44.0 [38.0–52.0] | 0.658 |
| Ⅲ P-wave amplitude (μV) | 55.0 [35.0–80.0] | 45.0 [33.8–71.3] | 57.5 [35.0–80.0] | 0.272 |
| Ⅲ QRS area(40 ms＊μV) | 31.0 [-347.0–287.0] | 31.0 [-180.0–168.0] | 33.0 [-379.0–294.3] | 0.952 |
| Ⅲ R-wave amplitude (μV) | 295.0 [130.0–557.5] | 295.0 [125.0–422.5] | 295.0 [133.8–570.0] | 0.571 |
| Ⅲ R-wave duration (msec) | 42.0 [26.0–57.0] | 42.0 [27.0–56.0] | 42.0 [26.0–58.0] | 0.863 |
| Ⅲ S-wave amplitude (μV) | 305.0 [155.0–725.0] | 215.0 [152.5–527.5] | 310.0 [162.5–818.8] | 0.523 |
| Ⅲ S-wave duration (msec) | 48.8 (±28.7) | 42.6 (±19.0) | 49.9 (±30.0) | 0.310 |
| Ⅲ ^*^STJ (μV) | -4.5 (±54.4) | -13.6 (±56.8) | -3.0 (±54.0) | 0.368 |
| Ⅲ ST MID (μV) | 10.0 [-15.0–33.8] | 0.0 [-10.0–25.0] | 10.0 [-15.0–35.0] | 0.568 |
| Ⅲ ST END (μV) | 15.0 [-20.0–50.0] | 0.0 [-10.0–35.0] | 20.0 [-25.0–50.0] | 0.523 |
| Ⅲ T-wave amplitude (μV) | 59.8 (±181.6) | 3.1 (±210.9) | 69.1 (±175.4) | 0.099 |
| Ⅲ T-wave amplitude modulation (μV) | 8.4 (±143.1) | -20.4 (±185.3) | 13.3 (±134.8) | 0.278 |
| Ⅲ Ventricular activation time (ms) | 40.0 [22.0–52.0] | 34.0 [15.0–46.0] | 40.0 [24.0–52.0] | 0.204 |
| aVR P’-wave amplitude (μV) | -70.0 [-95.0–-47.5] | -55.0 [-82.5–-40.0] | -70.0 [-95.0–-50.0] | 0.092 |
| aVR Q-wave amplitude (μV) | 489.5 (±260.0) | 522.9 (±263.7) | 483.5 (±260.2) | 0.545 |
| aVR Q-wave duration (msec) | 58.0 [44.0–74.0] | 48.0 [42.0–61.0] | 58.0 [46.0–78.5] | 0.080 |
| aVR Q-wave duration equivalent (msec) | 79.8 (±28.5) | 70.6 (±22.0) | 81.5 (±29.2) | 0.124 |
| aVR QRS area(40 ms＊μV) | -257.5 [-432.0–-89.0] | -300.0 [-450.0–-134.0] | -251.0 [-427.0–-85.0] | 0.443 |
| aVR R-wave amplitude (μV) | 95.0 [53.8–186.3] | 100.0 [52.5–165.0] | 95.0 [55.0–205.0] | 0.836 |
| aVR R-wave duration (msec) | 28.0 [16.0–46.0] | 22.0 [18.0–41.0] | 29.0 [16.0–49.5] | 0.828 |
| aVR ^*^STJ (μV) | 5.0 [-10.0–30.0] | 10.0 [0.0–40.0] | 5.0 [-10.0–30.0] | 0.317 |
| aVR ST MID (μV) | 0.0 [-25.0–20.0] | 0.0 [-25.0–40.0] | 0.0 [-25.0–20.0] | 0.348 |
| aVR ST END (μV) | -10.0 [-53.8–15.0] | 5.0 [-20.0–55.0] | -10.0 [-55.0–15.0] | 0.097 |
| aVR T-wave amplitude (μV) | -94.2 (±144.1) | -23.9 (±165.9) | -105.8 (±137.4) | **0.013** |
| aVR T-wave amplitude modulation (μV) | -91.3 (±106.5) | -55.5 (±122.9) | -97.4 (±102.7) | 0.081 |
| aVR Ventricular activation time (ms) | 64.0 [20.0–80.0] | 64.0 [27.5–73.5] | 64.0 [20.0–80.0] | 0.963 |
| aVL P-wave amplitude (μV) | 40.0 [30.0–55.0] | 35.0 [25.0–47.5] | 40.0 [30.0–57.5] | 0.188 |
| aVL QRS area (40 ms＊μV) | 139.5 [-57.3–408.0] | 103.0 [-6.0–103.0] | 157.0 [-75.0–424.0] | 0.911 |
| aVL R-wave amplitude (μV) | 457.7 (±341.1) | 400.0 (±287.6) | 467.3 (±349.1) | 0.362 |
| aVL R-wave duration (msec) | 54.0 (±28.9) | 48.4 (±23.5) | 55.0 (±29.7) | 0.294 |
| aVL ^*^STJ (μV) | 0.0 [-20.0–15.0] | 0.0 [-15.0–15.0] | 0.0 [-20.0–15.0] | 0.622 |
| aVL ST MID (μV) | -5.4 (±35.4) | -2.6 (±29.0) | -5.9 (±36.3) | 0.670 |
| aVL ST END (μV) | -5.0 [-30.0–20.0] | 5.0 [-20.0–20.0] | -5.0 [-30.0–20.0] | 0.500 |
| aVL T-wave amplitude (μV) | 2.4 (±132.8) | 30.9 (±126.8) | -2.5 (±133.7) | 0.267 |
| aVL T-wave amplitude modulation (μV) | -14.1 (±112.0) | 19.5 (±110.7) | -20.2 (±111.6) | 0.111 |
| aVL Ventricular activation time (ms) | 50.0 (±20.1) | 46.7 (±17.1) | 50.5 (±20.5) | 0.386 |
| aVF P-wave amplitude (μV) | 65.0 [50.0–95.0] | 67.5 [38.8–86.3] | 65.0 [50.0–95.0] | 0.414 |
| aVF QRS area (40 ms＊μV) | 144.5 [-184.8–373.0] | 173.0 [-81.0–365.0] | 144.0 [-210.0–373.0] | 0.797 |
| aVF R-wave amplitude (μV) | 144.5 [-184.8–373.0] | 372.5 [138.8–666.3] | 392.5 [206.3–653.8] | 0.735 |
| aVF R-wave duration (msec) | 55.6 (±26.3) | 50.7 (±20.4) | 56.4 (±27.1) | 0.322 |
| aVF S-wave amplitude (μV) | 237.5 [131.3–635.0] | 200.0 [125.0–340.0] | 260.0 [133.8–666.3] | 0.260 |
| aVF S-wave duration (msec) | 48.8 (±26.9) | 42.2 (±21.4) | 50.0 (±27.7) | 0.260 |
| aVF ^*^STJ (μV) | -5.0 [-35.0–15.0] | -5.0 [-30.0–10.0] | -5.0 [-35.0–20.0] | 0.376 |
| aVF ST MID (μV) | 10.0 [-15.0–30.0] | 5.0 [-15.0–25.0] | 10.0 [-15.0–30.0] | 0.333 |
| aVF ST END (μV) | 20.0 [-10.0–55.0] | 5.0 [-10.0–55.0] | 20.0 [-10.0–55.0] | 0.422 |
| aVF T-wave amplitude (μV) | 91.0 (±175.6) | 24.8 (±217.8) | 101.8 (±166.3) | 0.051 |
| aVF T-wave amplitude modulation (μV) | 50.0 [-47.0–120.0] | 35.0 [-86.8–101.3] | 55.0 [-45.0–120.0] | 0.377 |
| aVF Ventricular activation time (ms) | 43.7 (±16.9) | 38.8 (±13.8) | 44.5 (±17.3) | 0.125 |
| V1 P-wave amplitude (μV) | 40.0 [30.0–60.0] | 45.0 [35.0–65.0] | 40.0 [30.0–60.0] | 0.400 |
| V1 P’-wave amplitude (μV) | -55.0 [-85.0–-41.3] | -42.5 [-78.8–-33.8] | -55.0 [-85.0–-45.0] | 0.162 |
| V1 QRS area (40 ms＊μV) | -542.0  [-1019.0–-215.0] | -647.0  [-1006.0–-394.0] | -525.0  [-1027.0–-210.3] | 0.602 |
| V1 R-wave amplitude (μV) | 115.0 [55.0–275.0] | 127.5 [57.5–265.0] | 115.0 [55.0–275.0] | 0.883 |
| V1 R-wave duration (msec) | 24.0 [16.0–32.0] | 21.0 [17.5–30.0] | 24.0 [16.0–32.0] | 0.737 |
| V1 S-wave amplitude (μV) | 812.5 [551.3–1330.0] | 740.0 [537.5–1330.0] | 825.0 [552.5–1327.5] | 0.976 |
| V1 S-wave duration (msec) | 63.8 (±21.7) | 59.1 (±16.0) | 64.7 (±22.6) | 0.262 |
| V1 ^*^STJ (μV) | 55.0 [16.3–95.0] | 45.0 [5.0–95.0] | 55.0 [20.0–95.0] | 0.277 |
| V1 ST MID (μV) | 67.5 [26.3–115.0] | 50.0 [25.0–135.0] | 70.0 [30.0–115.0] | 0.669 |
| V1 ST END (μV) | 65.0 [16.3–138.8] | 60.0 [45.0–150.0] | 65.0 [15.0–135.0] | 0.794 |
| V1 T-wave amplitude (μV) | 115.0 [-35.0–225.0] | 145.0 [28.3–261.3] | 105.0 [-40.0–220.0] | 0.632 |
| V1 T-wave amplitude modulation (μV) | 25.0 [-93.8–100.0] | 65.0 [5.0–130.0] | 20.0 [-95.0–95.0] | 0.180 |
| V1 Ventricular activation time (ms) | 16.0 [12.0–26.0] | 16.0 [13.5–20.0] | 18.0 [12.0–26.0] | 0.429 |
| V2 P-wave amplitude (μV) | 45.0 [30.0–70.0] | 60.0 [30.0–72.5] | 45.0 [30.0–70.0] | 0.638 |
| V2 QRS area(40 ms＊μV) | -851.5  [-1642.0–-128.0] | -903.0  [-1265.0–-476.0] | -802.0  [-1708.0–-126.0] | 0.827 |
| V2 R-wave amplitude (μV) | 355.0 [170.0–632.5] | 395.0 [235.0–525.0] | 350.0 [165.0–645.0] | 0.733 |
| V2 R-wave duration (msec) | 30.0 [21.0–38.0] | 28.0 [22.0–34.0] | 30.0 [20.0–39.5] | 0.791 |
| V2 S-wave amplitude (μV) | 1533.3 (±1006.4) | 1465.0 (±1041.2) | 1547.3 (±1002.9) | 0.711 |
| V2 S-wave duration (msec) | 59.7 (±23.6) | 56.5 (±19.4) | 60.3 (±24.4) | 0.461 |
| V2 ^*^STJ (μV) | 75.0 [25.0–145.0] | 35.0 [20.0–100.0] | 85.0 [30.0–160.0] | 0.054 |
| V2 ST MID (μV) | 117.5 [55.0–218.8] | 95.0 [55.0–160.0] | 130.0 [55.0–220.0] | 0.192 |
| V2 ST END (μV) | 152.3 [76.3–295.0] | 125.0 [85.0–245.0] | 160.0 [75.0–305.0] | 0.308 |
| V2 T-wave amplitude (μV) | 320.0 [145.0–530.0] | 360.0 [220.0–410.0] | 315.0 [143.8–552.5] | 0.985 |
| V2 T-wave amplitude modulation (μV) | 125.0 [5.0–270.0] | 195.0 [65.0–280.0] | 105.0 [5.0–258.8] | 0.160 |
| V2 Ventricular activation time (ms) | 23.0 [16.0–30.0] | 22.0 [18.0–26.0] | 24.0 [16.0–34.0] | 0.411 |
| V3 P-wave amplitude (μV) | 50.0 [35.0–70.0] | 65.0 [52.5–70.0] | 50.0 [35.0–70.0] | 0.122 |
| V3 QRS area (40 ms＊μV) | -444.5  [-1223.3–197.3] | -140.0  [-629.0–212.0] | -481.0  [-1288.0–183.0] | 0.085 |
| V3 R-wave amplitude (μV) | 545.0 [262.5–1025.0] | 710.0 [405.0–1125.0] | 530.0 [250.0–997.5] | 0.091 |
| V3 R-wave duration (msec) | 38.0 [24.0–48.5] | 44.0 [32.0–50.0] | 36.0 [22.0–47.0] | 0.100 |
| V3 S-wave amplitude (μV) | 1190.0 [630.0–1713.8] | 1280.0 [775.0–1720.0] | 1170.0 [610.0–1690.0] | 0.668 |
| V3 S-wave duration (msec) | 56.8 (±24.7) | 53.4 (±17.6) | 57.4 (±25.7) | 0.457 |
| V3 ^*^STJ (μV) | 45.0 [5.0–85.0] | 15.0 [-30.0–40.0] | 50.0 [5.0–95.0] | **0.001** |
| V3 ST MID (μV) | 120.0 [40.0–180.0] | 60.0 [35.0–115.0] | 130.0 [45.0–185.0] | **0.019** |
| V3 ST END (μV) | 170.0 [60.0–265.0] | 95.0 [60.0–170.0] | 180.0 [60.0–270.0] | 0.084 |
| V3 T-wave amplitude (μV) | 297.5 [160.0–535.0] | 302.5 [113.8–483.8] | 297.5 [175.0–555.0] | 0.392 |
| V3 T-wave amplitude modulation (μV) | 148.0 [21.3–278.8] | 180.0 [60.0–260.0] | 130.0 [20.0–285.0] | 0.679 |
| V3 Ventricular activation time (ms) | 28.0 [21.0–38.0] | 28.0 [24.0–38.0] | 28.0 [20.0–38.0] | 0.685 |
| V4 P-wave amplitude (μV) | 55.0 [40.0–73.8] | 60.0 [45.0–70.0] | 50.0 [40.0–75.0] | 0.446 |
| V4 QRS area (40 ms＊μV) | 171.5 [-608.0–574.8] | 394.0 [37.0–820.0] | 133.0 [-669.0–497.0] | **0.028** |
| V4 R-wave amplitude (μV) | 1158.3 (±872.4) | 1781.2 (±1119.6) | 1054.5 (±781.5) | **<0.001** |
| V4 R-wave duration (msec) | 45.2 (±16.7) | 48.1 (±11.2) | 44.7 (±17.4) | 0.355 |
| V4 S-wave amplitude (μV) | 825.0 [465.0–1242.5] | 870.0 [505.0–1190.0] | 797.5 [451.3–1260.0] | 0.835 |
| V4 S-wave duration (msec) | 48.0 [37.0–65.0] | 46.0 [38.0–56.0] | 49.0 [36.5–68.0] | 0.494 |
| V4 ^*^STJ (μV) | -2.5 [-43.8–40.0] | -40.0 [-90.0–10.0] | 0.0 [-35.0–45.0] | **0.004** |
| V4 ST MID (μV) | 55.0 [0.0–115.0] | 15.0 [-50.0–65.0] | 55.0 [5.0–120.0] | **0.017** |
| V4 ST END (μV) | 85.0 [6.3–173.8] | 60.0 [-30.0–185.0] | 90.0 [15.0–170.0] | 0.261 |
| V4 T-wave amplitude (μV) | 192.5 [28.8–422.5] | 122.5 [-157.5–438.8] | 205.0 [51.3–408.8] | 0.148 |
| V4 T-wave amplitude modulation (μV) | 67.5 (±333.1) | -20.0 (±466.1) | 81.9 (±305.5) | 0.157 |
| V4 Ventricular activation time (ms) | 38.0 [28.0–44.5] | 40.0 [32.0–44.0] | 36.0 [26.0–45.0] | 0.413 |
| V5 P-wave amplitude (μV) | 50.0 [40.0–70.0] | 60.0 [37.5–65.0] | 50.0 [40.0–70.0] | 0.951 |
| V5 QRS area (40 ms＊μV) | 171.5 [-608.0–574.8] | 805.0 [565.0–1489.0] | 516.0 [75.0–940.0] | **0.011** |
| V5 R-wave amplitude (μV) | 1403.1 (±920.2) | 2098.6 (±1145.8) | 1289.5 (±828.3) | **<0.001** |
| V5 R-wave duration (msec) | 48.0 [40.0–64.0] | 50.0 [44.0–60.0] | 48.0 [40.0–64.0] | 0.564 |
| V5 S-wave amplitude (μV) | 370.0 [185.0–622.5] | 355.0 [217.5–487.5] | 370.0 [185.0–626.3] | 0.828 |
| V5 S-wave duration (msec) | 42.0 [30.0–56.0] | 38.0 [31.0–46.0] | 44.0 [30.0–58.0] | 0.254 |
| V5 ^*^STJ (μV) | -35.0 [-73.8–0.0] | -50.0 [-1000.0–-5.0] | -35.0 [-65.0–0.0] | 0.116 |
| V5 ST MID (μV) | -9.5 (±99.7) | -43.0 (±137.8) | -4.2 (±91.6) | 0.071 |
| V5 ST END (μV) | 12.6 (±150.8) | -28.8 (±186.7) | 19.1 (±143.9) | 0.140 |
| V5 T-wave amplitude (μV) | 76.1 (±337.8) | -11.7 (±416.7) | 90.0 (±323.1) | 0.171 |
| V5 T-wave amplitude modulation (μV) | -3.6 (±283.5) | -79.4 (±355.2) | 9.0 (±269.2) | 0.149 |
| V5 Ventricular activation time (ms) | 42.0 [36.0–52.0] | 42.0 [38.0–48.0] | 42.0 [36.0–54.0] | 0.609 |
| V6 P-wave amplitude (μV) | 55.0 [40.0–70.0] | 50.0 [30.0–67.5] | 55.0 [40.0–70.0] | 0.401 |
| V6 QRS area (40 ms＊μV) | 563.5 [281.5–1040.8] | 644.0 [452.0–1262.0] | 537.0 [254.0–971.0] | 0.102 |
| V6 R-wave amplitude (μV) | 1139.9 (±686.6) | 1539.8 (±904.6) | 1074.9 (±624.3) | **0.002** |
| V6 R-wave duration (msec) | 56.0 [44.0–74.0] | 56.0 [46.0–62.0] | 56.0 [40.0–76.0] | 0.841 |
| V6 S-wave amplitude (μV) | 195.0 [90.0–337.5] | 185.0 [102.5–390.0] | 200.0 [72.0–335.0] | 0.525 |
| V6 S-wave duration (msec) | 38.0 [28.0–58.0] | 39.0 [30.0–45.5] | 38.0 [26.0–58.0] | 0.892 |
| V6 ^*^STJ (μV) | -25.0 [-55.0–0.0] | -30.0 [-75.0–5.0] | -25.0 [-50.0–0.0] | 0.465 |
| V6 ST MID (μV) | -20.1 (±73.9) | -45.0 (±105.4) | -16.1 (±67.2) | 0.069 |
| V6 ST END (μV) | -5.3 (±108.7) | -30.4 (±136.5) | -1.3 (±103.5) | 0.214 |
| V6 T-wave amplitude (μV) | 46.1 (±244.8) | -2.8 (±286.1) | 53.7 (±238.0) | 0.304 |
| V6 T-wave amplitude modulation (μV) | -4.7 (±214.5) | -74.6 (±259.9) | 6.6 (±204.9) | 0.085 |
| V6 Ventricular activation time (ms) | 46.0 [40.0–56.0] | 44.0 [38.0–52.0] | 46.0 [40.0–58.0] | 0.382 |

**Supplemental Table S3**. Feature importance score and mean absolute SHAP value of each factor on train-validation data

We used the feature importance score and the mean absolute SHapley Additive exPlanation (SHAP) value to create a predictive model of inappropriate implantable cardioverter–defibrillator therapy. Each was added, multiplied by 10, and assigned the closest value in increments of 0.5. We then set a positive or negative score based on the positive or negative correlation. * cardiac resynchronization therapy; † diabetes mellites, ‡ ischemic cardiomyopathy.

|  | **Feature importance score** | **Mean absolute SHAP value** | **Sum of the scores multiplied by 10** | **Positive/ Negative correlation** | **Assigned point** |
| --- | --- | --- | --- | --- | --- |
| History of atrial arrhythmia | 0.352 | 0.210 | 5.629 | + | +5.5 |
| ^*^ CRT | 0.057 | 0.078 | 1.352 | – | -1.5 |
| V3 ST level at J point  ≥ 20 μV | 0.075 | 0.028 | 1.031 | + | +1.0 |
| V5 R-wave amplitude  ≥ 1400 μV | 0.073 | 0.040 | 1.125 | + | +1.0 |
| ^‡^ICM | 0.047 | 0.048 | 0.952 | – | -1.0 |
| ^†^DM | 0.028 | 0.039 | 0.673 | – | -0.5 |

**Supplemental Table S4.** Results of first screening for 14 machine learning models on train-validation data.

The result of the first screening by PyCaret with each default hyperparameters. All 14 models were simultaneously built and compared by PyCaret with 10-fold cross validation on 116 train-validation dataset, using all 16 parameters and default hyperparameter. All results of the models on train-validation data were described. Data was sorted by area under the receiver operating characteristic curve. * accuracy; † area under the receiver operating characteristic curve; ‡ F1-score (harmonic mean of precision and recall); § no available; || negative predictive value; # positive predictive value (identical to Precision); ** sensitivity (identical to Recall), *** specificity

| Model | ^†^AUROC | ^*^ACC | ^***^Spec. | ^**^Sens. (Recall) | ^#^PPV (Prec.) | \|\|NPV | ^‡^F1 |
| --- | --- | --- | --- | --- | --- | --- | --- |
| Extra Trees Classifier | 0.908 | 0.872 | 0.951 | 0.400 | 0.450 | 0.910 | 0.400 |
| Gradient Boosting Classifier | 0.892 | 0.846 | 0.893 | 0.600 | 0.475 | 0.933 | 0.490 |
| CatBoost Classifier | 0.878 | 0.846 | 0.893 | 0.550 | 0.475 | 0.933 | 0.457 |
| Extreme Gradient Boosting | 0.866 | 0.864 | 0.923 | 0.500 | 0.475 | 0.928 | 0.440 |
| Light Gradient Boosting Machine | 0.859 | 0.847 | 0.913 | 0.450 | 0.325 | 0.920 | 0.340 |
| Random Forest Classifier | 0.847 | 0.864 | 0.933 | 0.450 | 0.325 | 0.923 | 0.340 |
| Logistic Regression | 0.777 | 0.771 | 0.793 | 0.700 | 0.360 | 0.936 | 0.451 |
| Ada Boost Classifier | 0.771 | 0.838 | 0.892 | 0.500 | 0.420 | 0.924 | 0.420 |
| Linear Discriminant Analysis | 0.769 | 0.761 | 0.783 | 0.650 | 0.312 | 0.941 | 0.397 |
| Naive Bayes | 0.745 | 0.812 | 0.853 | 0.550 | 0.425 | 0.927 | 0.450 |
| Decision Tree Classifier | 0.626 | 0.828 | 0.902 | 0.350 | 0.250 | 0.907 | 0.280 |
| K Neighbors Classifier | 0.568 | 0.587 | 0.570 | 0.700 | 0.176 | 0.944 | 0.278 |
| Dummy Classifier | 0.500 | 0.715 | 0.800 | 0.200 | 0.025 | ^§^NA | 0.044 |
| Quadratic Discriminant Analysis | 0.450 | 0.799 | 0.900 | 0.100 | 0.017 | ^§^NA | 0.029 |
